# Examining the factor structure and validity of the WHOQOL-AGE among the oldest-old Chinese in Singapore

**DOI:** 10.3389/fpubh.2023.1119893

**Published:** 2023-10-02

**Authors:** Grand H.-L. Cheng, QiuShi Feng, Yap-Seng Chong, Woon-Puay Koh

**Affiliations:** ^1^Department of Medicine, Yong Loo Lin School of Medicine, National University of Singapore, Singapore, Singapore; ^2^Department of Sociology, National University of Singapore, Singapore, Singapore; ^3^Department of Obstetrics and Gynaecology, Yong Loo Lin School of Medicine, National University of Singapore, Singapore, Singapore; ^4^Singapore Institute for Clinical Sciences, Agency for Science Technology and Research (A*STAR), Singapore, Singapore; ^5^Healthy Longevity Translational Research Programme, Yong Loo Lin School of Medicine, National University of Singapore, Singapore, Singapore

**Keywords:** WHOQOL-AGE, quality of life, factor analysis, validity, oldest-old, Chinese, Singapore

## Abstract

**Objectives:**

A short measure of quality of life in old age is essential. The present study examined the factor structure and validity of the 13-item WHOQOL-AGE among the oldest-old.

**Methods:**

Data came from 1,000 Chinese aged ≥85 years in Singapore. Exploratory and confirmatory factor analyzes were conducted on the WHOQOL-AGE. Regression examined the demographic, social and health correlates of the identified factors.

**Results:**

Factor analyzes suggested a bifactor model of the WHOQOL-AGE, which comprised three specific factors, namely “health,” “environment” and “mastery,” in addition to the general factor (“overall”). Partial scalar invariance (concerning only one item) and scalar invariance were established across gender and education level respectively, generally supporting the measurement invariance of this model. Regression results demonstrated known-groups validity. Health correlates were more predictive of “health” than “environment” and “mastery,” with more basic and instrumental activities of daily living, lower depressive symptomatology and fewer falls positively relating to “health.” Strength of social network and social engagement (social correlates) positively related to “environment” and “mastery” but not “health.”

**Conclusion:**

The WHOQOL-AGE exhibits a bifactor structure and known-groups validity among the oldest-old Chinese in Singapore. It seems useful to capture different facets of quality of life in the concerned population.

## Introduction

The world’s older population grows at an unprecedented rate. In their old age, individuals may encounter health adversities including comorbidity and limitations in activities of daily living, and be disengaged from productive activities and social networks. Despite such challenges and adversities, they may still be satisfied with their conditions (1). Against this backdrop, assessment of quality of life (QoL) among older people has been regarded as an important subject in public health (2).

QoL is coined by the World Health Organization (WHO) as “individuals’ perception of their position in life in the context of the culture and value systems in which they live and in relation to their goals, expectations, standards and concerns” (3). The WHO has been advocating research on QoL, and initiatives include the 100-item WHOQOL-100 (3, 4) and its shorter version – the 26-item WHOQOL-BREF (5, 6). Subsequently, the EUROHIS-QOL 8-item index (7) has been derived based on the WHOQOL-100 and the WHOQOL-BREF. These instruments are generic measures of QoL and its applicability to older adults are questionable. For instance, they do not highlight main concerns in later life such as sensory abilities and autonomy. To address this problem, the 24-item WHOQOL-OLD has been developed with a focus on such concerns (8).

Nevertheless, the WHOQOL-OLD as a supplementary module needs to be administered together with the WHOQOL-BREF, and this combination seems too lengthy and overburdening to the more-impaired older respondents and imposes difficulties to assessment practices. Therefore, adapting the EUROHIS-QOL 8-item index (7) and the WHOQOL-OLD 6-item short form version 1 (9), researchers have advanced the 13-item WHOQOL-AGE as a standalone, time-saving measure of QoL of the older population (10).

To our knowledge, there exist three published studies of the factor structure of the WHOQOL-AGE. The first study was conducted on Europeans aged 18+ (10). Briefly, confirmatory factor analysis (CFA) identified a second-order model in which a second-order factor oversaw two first-order factors (Q1-Q8 on factor 1, items Q1 and Q9-Q13 on factor 2). Subsequent studies involved Europeans aged 18+ (11) and Chinese Taiwanese aged 70+ (2). Their CFA findings supported a bifactor model that incorporated two specific factors (Q1-Q8 on factor 1, items Q9-Q13 on factor 2) in addition to the general factor (that comprised all items). The methodological explanation is that specific factor 1 items adopt a bipolar rating format whereas factor 2 items adopt a unipolar format. Another explanation is that factor 1 items seem to capture satisfaction in personal asset while factor 2 items seem to capture self-efficacy in activities of daily living (2).

The knowledge of the factor structure of the WHOQOL-AGE remains problematic. Q1 (“How would you rate your quality of life?”), which is a generic item of QoL (11), should only load on the general factor but not on any specific factor with non-generic items (4). Moreover, the conceptual meanings of the specific factors are unclear. One factor assesses health (e.g., Q2) and external concerns including social relationships (Q6) and living conditions (Q7). Similarly, another factor assesses internal [e.g., autonomy (Q10)] as well as external [e.g., social relationships (Q13)] aspects. Of note, both factors cover social relationships. Perhaps due to these issues, these two factors were merely vaguely named as “factor 1” and “factor 2.” Furthermore, there is a lack of examination on the correlates of these two factors (10, 12–14).

The scale development literature dictates that the applicability of a measure is limited unless its factor structure is clear and its factors are interpretable and meaningful (15). In addition, the factors should show empirical specificity and are more or less differentially related to different correlates (16). With these principles in mind, here, we investigate the factor structure and validity of the WHOQOL-AGE. Compared with other model types (e.g., second-order models), bifactor models offer a number of advantages, one of which is recognizing multidimensionality while maintaining a unidimensional structure (17, 18). With the establishment of a bifactor model, researchers and practitioners can refer to the total score (general factor) and the scores of specific factors. Our first objective is thus, via factor analytic procedures, to establish a bifactor model of the WHOQOL-AGE, in which Q2 to Q13 load on the specific factors while all items including Q1 embed in the general factor (4). We study the measurement invariance of this model across gender and education level, two fundamental personal attributes that may shape interpretation of survey items (2).

Existing validation studies of the WHOQOL-Age have focused on its total score. For instance, concerning known-groups validity, individuals with chronic conditions were found to report a lower WHOQOL-AGE total score (10). Thus far, data on validity associated with the specific factors of the WHOQOL-AGE remains lacking. We are motivated to fill this gap because these data help ascertain factor structure and meaning (16). Our second objective is to revisit the known-groups validity of the WHOQOL-AGE. Following the usual practice in the QoL research (12, 19), we examine how demographic, social and health correlates relate to the identified factors of the WHOQOL-AGE. Pending better understanding of the specific factors, it is virtually illegitimate to detail formal hypotheses for the corresponding known-groups validity. Nevertheless, a reasonable speculation is, for example, that healthier (such as lower depressive symptomatology, fewer falls) individuals should score higher on the “health” factor (if found) than the less healthy counterparts. Moreover, health correlates should play a stronger role in the “health” factor than in other specific factors (19).

The two research objectives were addressed with a sample of Chinese aged ≥85 years in Singapore. The number of people aged 80+ is projected to increase threefold from 2019 to 2050 (20). South-East Asia is one of the regions where the oldest-old age group shows the largest percentage increases. It is meaningful to study QoL of the oldest-old (21), especially in South-East Asia. Thus far, research on the factor structure and validity WHOQOL-AGE exclusively among the oldest-old is lacking. Besides, this measure has rarely been scrutinized in Asian samples (2). Sociocultural and language issues may shape response patterns and affect scale development findings. It is desirable to evaluate factor structure and validity of a measure in samples with different backgrounds (11, 22). In this regard, the present examination of the WHOQOL-AGE among the oldest-old Chinese in Singapore is informative.

## Methods

### Sample

We utilized data from the SG90 study, which was nested within the Singapore Chinese Health Study (SCHS). The SCHS is a population-based prospective cohort study that examines genetic, lifestyle and environmental factors of common chronic diseases among middle-aged and older Chinese in Singapore (23). At the baseline of the SCHS (1993–1998), 63,275 individuals (27,959 men and 35,298 women) aged 45 to 74 years were recruited via in-person interviews. The participants belonged to either of the two major Chinese dialect groups in Singapore (Hokkiens or Cantonese), and were residents in public housing estates where over 85% of the local population lived during the recruitment period. Of the baseline participants, 52,322, 39,528, and 17,107 were re-contacted and took part in the first (1999–2004), second (2006–2010) and third (2014–2016) follow-up interviews, respectively. The SCHS and the follow-up studies were endorsed by the Institutional Review Board at the National University of Singapore (approval number H-17-027). All participants provided informed consent.

The SG90 (2017–2018) was established to examine health and psychosocial conditions of the oldest-old aged 85 years and above. Due to limited resources and funding, this study could only recruit 1,000 individuals. The SG90 was nested within the SCHS cohort, and among the participants of the SCHS, the SG90 recruited from 9,323 surviving individuals who were aged 85+ from 2017 to 2018. The SG90 participants were recruited on a rolling basis – of the first 1,501 individuals invited, the first 1,000 consenters (67%) were enrolled and interviewed face-to-face. Many of the constructs studied here, including the WHOQOL-AGE and correlates, were covered only in the SG90 but not in the previous waves of the SCHS.

The demographic record revealed that the SG90 participants were more likely to have received formal education than those from the SCHS who were age-eligible and alive, but were not included in the SG90 (58.4% vs. 53.0%; *p* = 0.010). Nevertheless, the difference was negligible (Cramer’s *V* = 0.04). Similarly, although the SG90 participants were younger at recruitment than the individuals who were not included in SG90 (*p* < 0.001), the age difference was negligible (65.8 ± 2.8 vs. 66.5 ± 3.2 years; Cohen’s *d* = 0.21). Besides, there was no group difference in gender composition (*p* = 0.307; Cramer’s *V* = 0.02). Demographically, these two groups of people were largely similar.

A sample of 1,000 individuals was sufficient for factor analysis (15, 16, 24). Due to missing data, the sample size for regression analysis was 979 (97.9%). This sample size offered enough power for regression with a small *F^2^*, a power level of 0.90 and an alpha level of 0.05 (25).

### Measures

#### Main measure

The WHOQOL-AGE contains 13 items (10, 11): (Q1) “How would you rate your quality of life?,” (Q2) “How satisfied are you with your hearing vision or other senses overall?,” (Q3) “How satisfied are you with your health?,” (Q4) “How satisfied are you with yourself?,” (Q5) “How satisfied are you with your ability to perform your daily living activities?,” (Q6) “How satisfied are you with your personal relationships?,” (Q7) “How satisfied are you with the conditions of your living place (your home)?,” (Q8) “How satisfied are you with the way you use your time?,” (Q9) “Do you have enough energy for everyday life?,” (Q10) “How much control do you have over the things you like to do?,” (Q11) “To what extent are you satisfied with your opportunities to continue achieving in life?,” (Q12) “Do you have enough money to meet your needs?,” and (Q13) “How satisfied are you with your intimate relationships in your life?” All items were assessed on a 5-point Likert scale, with higher values denoting better QoL (e.g., 1 = very dissatisfied, 5 = very satisfied for Q2; 1 = not at all, 5 = completely for Q9). The items in Chinese used here are comparable to those prepared in Taiwan (26).

#### Correlates

Following the QoL literature (12, 19) and given data availability, we considered 19 constructs as potential correlates of the WHOQOL-AGE. Demographic correlates included age, gender, highest level of education (no formal education vs. primary or above), housing type (1–2 room public vs. 3 room public vs. ≥ 4 room public or private; an indicator of socioeconomic status in Singapore), and financial adequacy. Financial adequacy was the perception of having sufficient financial resources to pay for medical expenses (adequate vs. inadequate).

Regarding social correlates, indicators of social connectedness included marital status (married vs. single/ divorced/ widowed), living arrangement (living with others vs. living alone), and strength of social network (composite score of 2 items on structural aspect and 2 items on functional aspect) (27). Measures of social behavior included social engagement and productive engagement (less than once a month vs. at least once a month but less than once a week vs. at least once a week). Social engagement captured participation in social activities, participation in senior club events and attendance in a place of worship, and productive engagement referred to paid employment, volunteering and housework.

Health correlates covered health status and health behavior. Concerning health status, one indicator was self-reported comorbidity (diabetes, stroke, cancer, acute myocardial infarction, heart failure, kidney failure, chronic respiratory lung disease, Parkinson’s disease). Basic activities of daily living (BADL) were assessed with the 10-item Barthel ADL Index (28). Instrumental activities of daily living (IADL) were captured by 8 items (29). Depressive symptomatology was measured by the 15-item Geriatric Depression Scale (GDS-15; *α* = 0.82) (30). Cognitive status (intact vs. impaired) was evaluated by the 30-item Singapore-Modified Mini-Mental State Examination (SM-MMSE; *α* = 0.80) (31). On falls, participants reported how many times they had fallen in the past year (none vs. once vs. two or more). For health behavior, we studied exercise (e.g., jogging, Tai Chi; at least once a week vs. less than once a week), drinking alcohol (at least once a week vs. less than once a week), and current smoking (yes vs. no).

### Statistical analyses

#### Exploratory factor analysis

For objective 1, we first performed EFA (*N* = 1,000) to evaluate the factor structure of the WHOQOL-AGE. We had proposed a bifactor model of the WHOQOL-AGE. As mentioned, Q1 is a generic item of QoL (11) and should not load on any specific factor (4) of the model. Hence, Q1 was not incorporated in EFA. Number of factors was determined by parallel analysis (32). Upon the results of parallel analysis, principal factor analysis was conducted with oblique rotation (oblimin method). To interpret the factors, we attended to the rotated factor loadings>0.40 (16), and referred to the WHOQOL literature (5, 6, 8, 9).

#### Confirmatory factor analysis

Subsequently, we adopted CFA (*N* = 1,000) to test the bifactor model of the WHOQOL-AGE (see Discussion for the issues of using the same sample for EFA and CFA). While Q2 to Q13 loaded on the specific factors as suggested by EFA, all items including Q1 embedded in the general factor. The model was deemed acceptable if it met the following criteria: non-significant *X*^2^, comparative fit index (CFI) ≥ 0.90, root mean-square error of approximation (RMSEA) < 0.10, and standardized root mean-square residual (SRMR) < 0.10 (17). Vuong test of nonnested models (33) compared the current bifactor model with the alterative bifactor model and the second-order model suggested in previous studies (2, 10, 11).

We also implemented multigroup CFA (34) to examine whether the current bifactor model held across gender and education level (no formal education vs. primary or above) (i.e., measurement invariance). We consecutively tested configural invariance (invariance of patterns of factor loadings), metric invariance (invariance of values of factor loadings), and scalar invariance (invariance of both factor loadings and item intercepts). A significant decrease in model fit was indicated by significant ∆*X*^2^, ∆CFI ≤ −0.010, ∆RMSEA≥0.015, and ∆SRMR≥0.030 (17). If a certain invariance was not retained, we attempted to establish the relevant partial invariance based on modification indices. Although reported for record, *X*^2^ and ∆*X*^2^ should be less underscored due to their high sensitivity to sample size.

#### Regression

For objective 2, we conducted regression analysis (*N* = 979) to assess the known-groups validity of the WHOQOL-AGE. The scores (simple average of component items) of the specific and general factors of the WHOQOL-AGE were regressed on the aforementioned 19 demographic, social and health correlates: age, gender, education level, housing type, financial adequacy, marital status, living arrangement, strength of social network, social engagement, productive engagement, chronic diseases, BADL, IADL, depressive symptomatology, cognitive status, falls, exercise, drinking alcohol, and current smoking.

CFA was conducted by Mplus 8 and R packages “lavaan,” “semTools,” and “nonnest2.” EFA and regression were done with Stata 17.

## Results

Among the 1,000 participants, the mean (standard deviation) age was 87.9 ± 2.4 years (between 85 and 97 years; Supplementary Table S1). Three hundred fifty-three individuals were men and 647 were women. Four hundred fifty-six individuals had received no formal education and 544 had received at least primary education. Supplementary Table S2 reports the correlation among the 13 WHOQOL-AGE items. All correlations were positive and significant (*p* < 0.01).

### EFA findings

The KMO value was marvelous (0.90) and the Barlett’s sphericity test result was significant (*p* < 0.001), indicating that the sample size was sufficient and that the correlations between variables were overall significantly different from zero, respectively. Parallel analysis showed that the difference between actual data eigenvalues and random data eigenvalues remained positive up to the third factor (Supplementary Table S3), suggesting that there should emerge three specific factors of the WHOQOL-AGE. Accordingly, we specified three factors in principal factor analysis. Principal factor analysis revealed that Q2, Q3, Q4 and Q5 primarily loaded on the first factor (Table 1). These items refer to health issues. We thus labeled the first factor as “health.” Q6, Q7, Q8, Q11 and Q13 predominantly loaded on the second factor. These items concern social relationships, activities, and living conditions. The second factor was labeled as “environment.” Q9, Q10 and Q12 mainly embedded in the third factor. These items cover autonomy, capacity, and capital. Hence, the third factor was termed as “mastery.”

**Table 1:** Rotated factor loadings obtained from EFA.

	Factor 1	Factor 2	Factor 3
Q2	**0.51**	−0.01	0.05
Q3	**0.78**	−0.05	0.01
Q4	**0.62**	0.16	0.04
Q5	**0.48**	0.16	0.11
Q6	0.26	**0.43**	−0.10
Q7	0.00	**0.56**	0.02
Q8	−0.04	**0.68**	0.06
Q9	0.24	−0.02	**0.49**
Q10	−0.03	−0.01	**0.55**
Q11	0.23	**0.43**	0.08
Q12	−0.03	0.09	**0.53**
Q13	0.07	**0.41**	−0.00

As mentioned in the text, Q1 was not included here. Factor loadings > 0.40 are in bold.

### CFA findings

**Figure 1:**
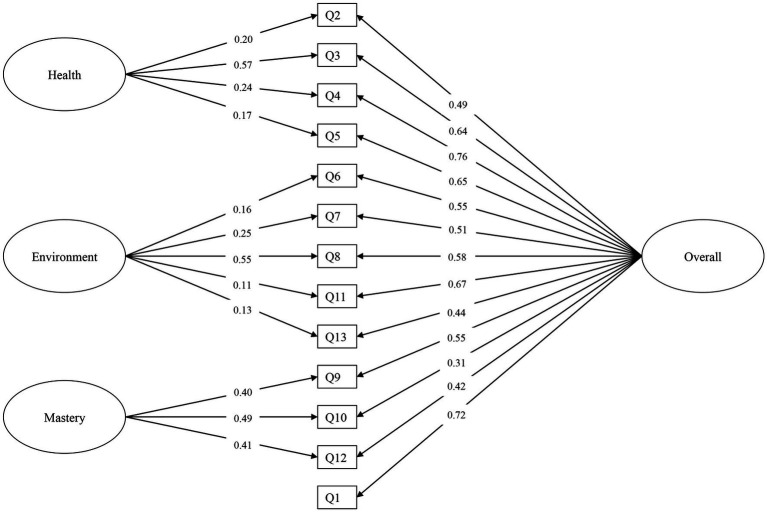
CFA results of the proposed bifactor model. Standardized factor loadings are shown. All factor loadings were significant at *p* < 0.05. Residual variances were all significant (not shown for simplicity; details available from the authors). Residual covariances were not added to the model.

In the present bifactor model of the WHOQOL-AGE, Q2 to Q13 loaded on the specific factors of “health,” “environment” or “mastery,” and all items including Q1 embedded in the general factor (termed “overall”). CFA showed that our model fitted the data well: CFI = 0.982, RMSEA = 0.036, SRMR = 0.024. As illustrated in Figure 1, all factor loadings were positive and significant (*p* < 0.05).

Separate CFA revealed that the second-order model suggested in a previous study (10) was acceptable (CFI = 0.919, RMSEA = 0.071, SRMR = 0.044). All factor loadings were positive and significant (Supplementary Figure S1). However, Vuong test indicated our bifactor model was significantly better than the second-order model (*Z* = 5.91, *p* < 0.001). The alternative bifactor model (2, 11) also fitted the data well (CFI = 0.983, RMSEA = 0.036, SRMR = 0.024). Vuong test indicated that in general, this alternative model did not fit worse than our bifactor model (*Z* = 0.22, *p* = 0.586). Nevertheless, unlike our model, not all factor loadings in the alternative model were positive and significant (Supplementary Figure S2). Taken together, our bifactor model was better than the second-order and the alternative bifactor counterparts.

Table 2 summaries the findings of measurement invariance of the proposed bifactor model. Concerning invariance across gender, configural invariance was supported with CFI (0.974), RMSEA (0.044) and SRMR (0.030) falling within recommended values. Metric invariance was also supported (∆CFI = 0.000, ∆RMSEA = −0.003, ∆SRMR = 0.011). ∆CFI (−0.012) suggested a rejection of scalar invariance, but with the constraints on Q13 intercept released, partial scalar invariance was achieved (∆CFI = −0.002, ∆RMSEA = −0.001, ∆SRMR = −0.001). Regarding invariance across education level, configural invariance was established in terms of CFI (0.973), RMSEA (0.045) and SRMR (0.031). Metric invariance (∆CFI = −0.007, ∆RMSEA = 0.001, ∆SRMR = 0.018) and scalar invariance (∆CFI = −0.008, ∆RMSEA = 0.003, ∆SRMR = −0.002) were also established. The proposed bifactor model generally held across gender and education level.

**Table 2:** Measurement invariance of the proposed bifactor model across gender and education level.

	*X*^2^ or (∆*X*^2^)	df or (∆df)	*p*	CFI or (∆CFI)	RMSEA or (∆RMSEA)	SRMR or (∆SRMR)
Gender (men vs. women)						
1. Configural	209.71	106	<0.001	0.974	0.044	0.030
2. Metric	231.93	127	<0.001	0.974	0.041	0.041
3. Scalar	286.26	136	<0.001	0.962	0.047	0.041
3a. Partial scalar ^#^	245.16	135	<0.001	0.972	0.040	0.040
2. vs. 1.	(22.22)	(21)	0.387	(0.000)	(−0.003)	(0.011)
3. vs. 2.	(54.33)	(9)	<0.001	(−0.012)	(0.006)	(0.000)
3a. vs. 2.	(13.23)	(8)	0.104	(−0.002)	(−0.001)	(−0.001)
Education level (no formal education vs. primary or above)						
1. Configural	213.65	106	<0.001	0.973	0.045	0.031
2. Metric	264.25	127	<0.001	0.966	0.046	0.049
3. Scalar	302.48	136	<0.001	0.958	0.049	0.047
2. vs. 1.	(50.60)	(21)	<0.001	(−0.007)	(0.001)	(0.018)
3. vs. 2.	(38.23)	(9)	<0.001	(−0.008)	(0.003)	(−0.002)

CFI, comparative fit index; RMSEA, root mean-square error of approximation; SRMR, standardized root mean-square residual. ^#^The intercept of Q13 was not constrained to be equal across gender.

### Regression findings

Following the factor analysis results, we computed the scores (simple average of component items) of “health,” “environment,” “mastery” and “overall” (Supplementary Table S4) for regression. Among the studied demographic correlates, only financial adequacy was a significant correlate of the WHOQOL-AGE. Specifically, the oldest-old who perceived themselves to have adequate financial resources reported higher scores on “health” (*B* = 0.15, *p* = 0.006) and “overall” (*B* = 0.11, *p* = 0.005; Table 3).

**Table 3:** Relation of demographic, social and health correlates with the specific and general factors of the WHOQOL-AGE.

	Health (*F* = 21.45, adj. *R^2^* = 0.31)	Environment (*F* = 10.55, adj. *R^2^* = 0.17)	Mastery (*F* = 16.55, adj. *R^2^* = 0.26)	Overall (*F* = 25.38, adj. *R^2^* = 0.35)
Demographic correlates				
Age	0.01	0.00	−0.01	0.00
Male gender	0.04	0.04	0.06	0.05
Primary education or above	−0.08	0.02	0.06	−0.00
Housing type (ref: 1–2 room public)				
3 room public	−0.05	−0.05	−0.01	−0.03
≥ 4 room public/private	−0.04	−0.06	−0.00	−0.03
Adequate financial resources	0.15**	0.08	0.08	0.11**
Social correlates				
Married	−0.02	0.00	0.04	−0.00
Living alone	−0.20**	−0.10	−0.01	−0.11*
Strength of social network	0.02	0.03*	0.09***	0.05**
Social engagement (ref.: less than once a month)				
At least once a month but less than once a week	0.02	−0.02	0.03	0.00
At least once a week	0.05	0.06	0.09*	0.07*
Productive engagement (ref.: less than once a month)				
At least once a month but less than once a week	−0.05	−0.01	−0.09	−0.05
At least once a week	−0.01	0.02	−0.06	−0.01
Health correlates				
Chronic diseases	0.01	0.03	0.01	0.02
BADL	0.02*	0.00	0.01	0.01
IDAL	0.05**	0.02	0.05**	0.03**
Depressive symptomatology	−0.10***	−0.06***	−0.08***	−0.08***
Intact cognition	−0.14**	−0.04	−0.07	−0.08**
Falls (ref.: none)				
Once	0.04	0.03	0.03	0.03
Twice or more	−0.17**	−0.01	−0.09	−0.09
Exercise at least once a week	−0.05	0.02	0.04	0.00
Drinking alcohol at least once a week	−0.08	−0.05	−0.18	−0.08
Current smoking	0.07	−0.05	−0.03	−0.01

The four regression models were significant with *p* < 0.001. Regression coefficients are reported. *p < 0.05, **p < 0.01, ***p < 0.001.

Regarding social correlates, living alone was linked with lower levels of “health” (*B* = −0.20, *p* = 0.007) and “overall” (*B* = −0.11, *p* = 0.033). Stronger social network was related to higher levels of “environment” (*B* = 0.03, *p* = 0.025), “mastery” (*B* = 0.09, *p* < 0.001) and “overall” (*B* = 0.05, *p* = 0.001). Social engagement at least once a week was associated with higher levels of “mastery” (*B* = 0.09, *p* = 0.040) and “overall” (*B* = 0.07, *p* = 0.034).

Concerning health correlates, depressive symptomatology was negatively associated with “health” (*B* = −0.10, *p* < 0.001), environment” (*B* = −0.06, *p* < 0.001), “mastery” (*B* = −0.08, *p* < 0.001) and “overall” (*B* = −0.08, *p* < 0.001). IADL was positively related to “health” (*B* = 0.05, *p* = 0.001), “mastery” (*B* = 0.05, *p* = 0.001) and “overall” (*B* = 0.03, *p* = 0.001). BADL (*B* = 0.02, *p* = 0.018) and falls (twice or more; *B* = −0.17, *p* = 0.009) were positively and negatively related to “health,” respectively. Cognitively intact was shown to be negatively linked with “health” (*B* = −0.14, *p* = 0.001) and “overall” (*B* = −0.08, *p* = 0.009).

## Discussion

Against population aging, a brief and time-saving measure of QoL in old age is essential. The present study examined the factor structure (objective 1) and known-groups validity (objective 2) of the 13-item WHOQOL-AGE with a sample of the oldest-old Chinese in Singapore.

The WHOQOL-AGE exhibited a bifactor model that comprised three specific factors, namely “health,” “environment” and “mastery,” in addition to the general factor (“overall”). “Health” (about physical and psychological health) and “mastery” (autonomy, capacity, capital) focused on internal aspects, whereas “environment” focused on external issues (social relationships, activities, living conditions). The WHOQOL-AGE is partly rooted in the WHOQOL-100 (3, 4) which incorporates four domains: physical, psychological, social, environment. The resemblance between the observed specific factors of the WHOQOL-AGE and the conception of the WHOQOL-100 makes good sense.

The specific factors of the WHOQOL-AGE here are different from those reported previously (2, 10, 11). One possible account is that recognizing Q1 as a generic item (11), we did not put it under any specific factor (4). Besides, we applied the WHOQOL-AGE exclusively to the oldest-old. Mastery refers to capacity for participation and contribution, and perceived independence and usefulness (35). Perhaps because mastery is an increasing concern with age (36), it is more explicit and more readily identified in the oldest-old. In any event, compared with those reported in previous research, the factors identified in the present study are more meaningful and interpretable. Our findings better articulate what elements of QoL the WHOQOL-AGE captures, and this naturally enhances practicality and facilitates usage of this instrument.

The present bifactor model of the WHOQOL-AGE showed scalar invariance across education level. It also demonstrated partial scalar invariance across gender, with the intercept of Q13 (“How satisfied are you with your intimate relationships in your life?”) unconstrained. Women place a greater emphasis on intimacy than men (37), but they are less likely to remain married (12% women vs. 70% men in our dataset) because they have longer life expectancy and greater likelihood of being widowed (38). This backdrop may lead to a gendered interpretation and processing of Q13. Nevertheless, Q13 was the only item which intercept could not be constrained across gender. The general picture is thus that the present bifactor model holds across gender as well as education level.

Turning to the known-groups validity of the WHOQOL-AGE, BADL and IADL were positive correlates, and depressive symptomatology and falls were negative correlates of the “health” factor. Demographic and social correlates also played a role in “health.” Our measure of financial adequacy concerned the perception of having sufficient financial resources to pay for medical expenses. This should be why there was a positive association between financial adequacy and “health.” Living alone is a driver for lower health-related QoL (39). Indeed, we found living alone as a negative correlate of “health.”

Affective state, as depicted by depressive symptomatology, may be linked with diverse aspects of QoL (40). In line with the literature, our data showed a negative relationship of depressive symptomatology with not only “health” but also “environment” and “mastery.” Our data also revealed that apart from “health,” IADL was positively associated with “mastery” (36), which refers to autonomy and capacity (35).

Strength of social network and social engagement did not relate to “health.” On the other hand, strength of social network, which speaks to social connectedness in household and non-household settings, positively related to “environment” and “mastery.” Social engagement was a positive correlate of “mastery,” congruent with the wisdom that active participation in social activities may entail stronger perceptions of independence and usefulness (41).

Our multivariate findings showed that intact cognition was negatively associated with “health” as well as “overall,” but note that on a bivariate level, cognitive status was actually uncorrelated with these two factors (*r* = 0.01, *p* = 0.641; *r* = 0.05, *p* = 0.146). These data collectively indicate the emergence of the suppressor effect, a statistical phenomenon that is difficult to interpret (42). Some past studies have documented that cognitive function is largely unrelated to QoL in the oldest-old age on a multivariate level (21, 43). Taken together, the observed negative linkage of intact cognition with “health” and “overall” should not be overinterpreted.

In summary, “health,” “environment,” and “mastery” were accounted for by different arrays of correlates, with “health” being linked with more health correlates than the latter factors (19). At the same time, virtually all significant correlates of “health,” “environment,” and “mastery” were associated with “overall.” These data demonstrate the uniqueness of the specific factors and the known-groups validity of our bifactor model of the WHOQOL-AGE. That said, we call for more validation data of the WHOQOL-AGE – for example, its relationship with the WHOQOL-BREF and WHOQOL-OLD (convergent validity).

There are some caveats associated with the present study. It is not uncommon that published scales show low reliability (44), but one may be concerned that the Cronbach’s alpha of “mastery” was merely 0.62. Cronbach’s alpha is a function of number of items and average correlation between pairs of items (44). “Mastery” only showed marginally acceptable reliability likely because it only involved three items, which were, nevertheless, moderately positively intercorrelated (*r* > 0.3, *p* < 0.01; Supplementary Table S2). Given that the intercorrelations were moderate in size, and that CFA findings strongly supported the present bifactor model of the WHOQOL-AGE, the low reliability of “mastery” should not have created much noise and not be too problematic (45).

It has been recommended that the sample-size to parameters ratio for CFA should be at least 10:1 (16, 24). Our sample size just fulfilled this rule of thumb for the testing of measurement invariance in the context of CFA. Hence, we did not conduct EFA and CFA on subsamples (e.g., *n* = 300 for EFA; *n* = 700 for CFA) (15), but performed them on the whole sample (16, 46, 47). The latter practice is important in its own right–if congruence is not observed between EFA and CFA on the same data, it is unlikely that CFA will corroborate EFA findings in a different sample (46, 48, 49). In any case, we call for replication studies for the factor structure of the WHOQOL-AGE reported here.

Another issue is that the participants were survivors in a longitudinal cohort with follow-up of about 25 years. One may argue that our findings are limited to the relatively healthy oldest-old. Replication studies that adopt representative samples are needed. We also acknowledge that regression analysis was conducted with cross-sectional data. To develop better knowledge of known-groups validity, future research should analyze temporal causations with longitudinal data. In addition, scholars should study other forms of validity such as convergent validity and predictive validity.

This study examined the WHOQOL-AGE in Singapore, an Asian context. Our results suggested a good potential of the WHOQOL-AGE to be applied in other Asian societies, though accumulated investigations of this instrument in other regions and populations are surely needed. The cultural equivalence of the translated WHOQOL-AGE deserves more discussions. Singaporeans as Asians may be less likely to use the extreme values on scales than the Westerners (50). Besides, Singapore emphasizes family values. Our sample of the oldest-old Chinese in Singapore may consider the item on intimate relationships (Q13) of the WHOQOL-Age as sensitive information that they were unwilling to share with strangers. On the item about living conditions (Q7), all respondents were residents in public housing estates in Singapore at recruitment, and this may limit the heterogeneity in their responses to this item. Notably, nearly half of the studied oldest-old did not receive formal education. Hence, although the SG90 questionnaires were supposed to be administered in Mandarin during interviews, most of the interviews were conducted in the Hokkien or Cantonese dialect as a workaround, since the Chinese in this cohort were restricted to these two major dialect groups of Chinese in Singapore. Another language concern is that the Mandarin or dialects used in Singapore is not entirely the same as those used in Mainland China and Taiwan. Due to these sociocultural and language issues, our observations in the oldest-old Chinese in Singapore should not be mechanistically assumed to be generalizable to other samples.

## Conclusion

The 13-item WHOQOL-AGE exhibits a bifactor structure and known-groups validity among the oldest-old Chinese in Singapore. Depending on their objectives, scholars and practitioners may use the whole WHOQOL-AGE scale (i.e., general factor), or focus on its subscales (specific factors). With good properties, flexibility and parsimony, the WHOQOL-AGE seems a promising measure of QoL in the concerned population.

## Data availability statement

The raw data supporting the conclusions of this article will be made available by the authors, without undue reservation.

## Ethics statement

The studies involving humans were approved by Institutional Review Board at the National University of Singapore (approval number H-17-027). The studies were conducted in accordance with the local legislation and institutional requirements. The participants provided their written informed consent to participate in this study.

## Author contributions

GC designed the research, analyzed the data, and drafted the paper. QF provided feedback on the data analysis and result interpretation, and contributed to revising the paper. Y-SC and W-PK administered the data collection, and contributed to revising the paper. All authors contributed to the article and approved the submitted version.
