# Supplementary material for: Examining the factor structure and validity of the WHOQOL-AGE among the oldest-old Chinese in Singapore
Source: Front Public Health. 2023 Oct 2;11:1119893. doi: 10.3389/fpubh.2023.1119893 (PMC10581834; doi:10.3389/fpubh.2023.1119893)
Supplement: Supplementary file 1 [file Data_Sheet_1.docx]

***Supplementary Material***

**Examining the factor structure and validity of the WHOQOL-AGE among the oldest-old Chinese in Singapore**

**Grand H.-L. Cheng *, QiuShi Feng, Yap-Seng Chong, Woon-Puay Koh**

*** Correspondence:** Grand H.-L. Cheng: grand.cheng@yahoo.com

**Supplementary Table 1.** Descriptive statistics of the WHOQOL-Age items and correlates

| Variable | *M* (*SD*) or % |
| --- | --- |
| WHOQOL-AGE (range: 1–5) |  |
| Q1 | 3.6 (0.8) |
| Q2 | 3.6 (0.9) |
| Q3 | 3.5 (0.9) |
| Q4 | 3.7 (0.9) |
| Q5 | 3.6 (0.9) |
| Q6 | 3.7 (0.8) |
| Q7 | 4.1 (0.7) |
| Q8 | 3.9 (0.7) |
| Q9 | 3.2 (0.9) |
| Q10 | 3.0 (1.0) |
| Q11 | 3.7 (0.8) |
| Q12 | 3.1 (0.9) |
| Q13 | 3.7 (0.9) |
| Demographic correlates |  |
| Age (range: 85–97) | 87.9 (2.4) |
| Gender |  |
| Women | 64.7 |
| Men | 35.3 |
| Education level |  |
| No formal education | 45.6 |
| Primary or above | 54.4 |
| Housing type |  |
| 1-2 room public | 8.8 |
| 3 room public | 35.5 |
| ≥ 4 room public/ private | 55.7 |
| Financial adequacy |  |
| Inadequate | 15.9 |
| Adequate | 84.1 |
| Social correlates |  |
| Marital status |  |
| Single/ divorced/ widowed | 67.4 |
| Married | 32.6 |
| Living arrangement |  |
| Living with others | 90.3 |
| Living alone | 9.7 |
| Strength of social network (range: 0–4) | 2.9 (1.0) |
| Social engagement |  |
| Less than once a month | 53.8 |
| At least once a month but less than once a week | 12.5 |
| At least once a week | 33.7 |
| Productive engagement |  |
| Less than once a month | 21.9 |
| At least once a month but less than once a week | 7.9 |
| At least once a week | 70.2 |

| Variable | *M* (*SD*) or % |
| --- | --- |
| Health correlates |  |
| Chronic diseases (range: 0–8) | 0.6 (0.7) |
| BADL (range: 0–20) | 18.8 (2.7) |
| IDAL (range: 0–8) | 6.1 (2.1) |
| Depressive symptomatology (range: 0–15) | 4.3 (3.4) |
| Cognitive status |  |
| Impaired | 27.2 |
| Intact | 72.8 |
| Falls |  |
| None | 76.7 |
| Once | 12.9 |
| Twice or more | 10.4 |
| Exercise |  |
| Less than once a week | 25.8 |
| At least once a week | 74.2 |
| Drinking alcohol |  |
| Less than once a week | 96.6 |
| At least once a week | 3.4 |
| Current smoking |  |
| No | 94.2 |
| Yes | 5.8 |

BADL= basic activities of daily living, IADL= instrumental activities of daily living.

**Supplementary Table 2.** Correlation among the WHOQOL-AGE items

| Item | Q1 | Q2 | Q3 | Q4 | Q5 | Q6 | Q7 | Q8 | Q9 | Q10 | Q11 | Q12 | Q13 |
| --- | --- | --- | --- | --- | --- | --- | --- | --- | --- | --- | --- | --- | --- |
| Q1 | - |  |  |  |  |  |  |  |  |  |  |  |  |
| Q2 | 0.33 | - |  |  |  |  |  |  |  |  |  |  |  |
| Q3 | 0.43 | 0.43 | - |  |  |  |  |  |  |  |  |  |  |
| Q4 | 0.54 | 0.42 | 0.62 | - |  |  |  |  |  |  |  |  |  |
| Q5 | 0.43 | 0.34 | 0.52 | 0.54 | - |  |  |  |  |  |  |  |  |
| Q6 | 0.39 | 0.29 | 0.36 | 0.42 | 0.41 | - |  |  |  |  |  |  |  |
| Q7 | 0.43 | 0.23 | 0.31 | 0.37 | 0.31 | 0.35 | - |  |  |  |  |  |  |
| Q8 | 0.44 | 0.26 | 0.33 | 0.44 | 0.40 | 0.40 | 0.44 | - |  |  |  |  |  |
| Q9 | 0.40 | 0.32 | 0.43 | 0.42 | 0.39 | 0.25 | 0.27 | 0.29 | - |  |  |  |  |
| Q10 | 0.16 | 0.16 | 0.20 | 0.25 | 0.28 | 0.13 | 0.12 | 0.21 | 0.36 | - |  |  |  |
| Q11 | 0.47 | 0.33 | 0.45 | 0.50 | 0.44 | 0.38 | 0.37 | 0.45 | 0.34 | 0.22 | - |  |  |
| Q12 | 0.32 | 0.22 | 0.26 | 0.31 | 0.26 | 0.16 | 0.22 | 0.24 | 0.39 | 0.33 | 0.29 | - |  |
| Q13 | 0.35 | 0.19 | 0.27 | 0.34 | 0.26 | 0.24 | 0.23 | 0.34 | 0.20 | 0.09 | 0.34 | 0.20 | - |

All correlations were significant at *p*< 0.01.

**Supplementary Table 3.** Results of parallel analysis

| Factor | Actual data eigenvalues | Random data eigenvalues | Difference in eigenvalues |
| --- | --- | --- | --- |
| 1 | 4.00 | 0.20 | 3.80 |
| 2 | 0.45 | 0.15 | 0.30 |
| 3 | 0.28 | 0.11 | 0.16 |
| 4 | 0.05 | 0.08 | -0.02 |
| 5 | 0.01 | 0.05 | -0.03 |
| 6 | -0.05 | 0.02 | -0.07 |
| 7 | -0.08 | -0.00 | -0.08 |
| 8 | -0.10 | -0.03 | -0.07 |
| 9 | -0.11 | -0.06 | -0.05 |
| 10 | -0.13 | -0.09 | -0.05 |
| 11 | -0.17 | -0.12 | -0.05 |
| 12 | -0.19 | -0.16 | -0.03 |

**Supplementary Table 4.** Descriptive statistics and intercorrelations of the specific and general factors of the WHOQOL-AGE

| Factor | | 1 | 2 | 3 | 4 |
| --- | --- | --- | --- | --- | --- |
| 1. | Overall | (0.86) |  |  |  |
| 2. | Health | 0.88*** | (0.78) |  |  |
| 3. | Environment | 0.85*** | 0.64*** | (0.72) |  |
| 4. | Mastery | 0.71*** | 0.49*** | 0.40*** | (0.62) |
| *M* | | 3.57 | 3.61 | 3.81 | 3.10 |
| *SD* | | 0.53 | 0.72 | 0.54 | 0.72 |

Reliabilities (Cronbach’s alpha) are in parentheses. *** *p*< 0.001.


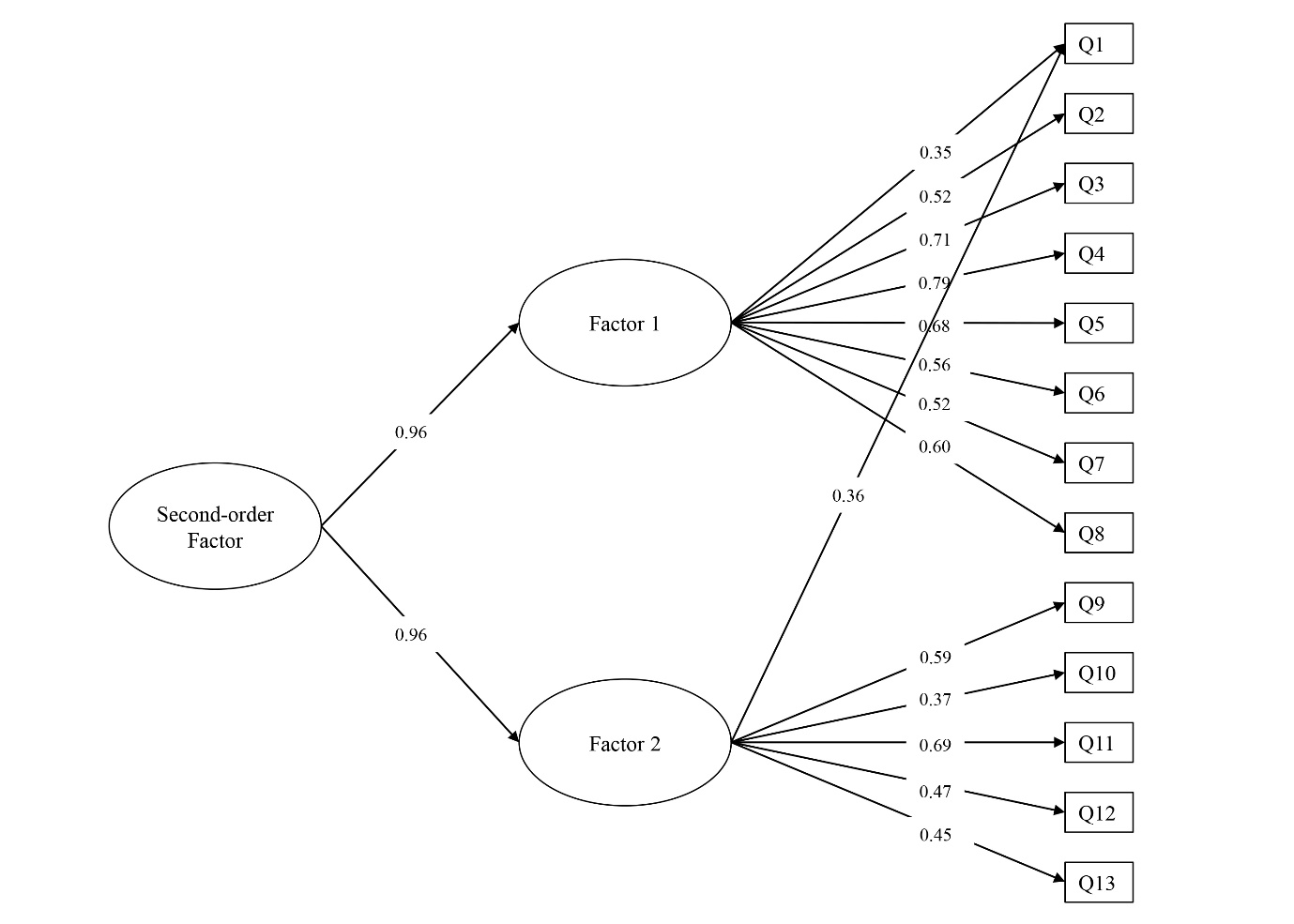


**Supplementary Figure 1.** CFA results of the second-order model (1). Standardized factor loadings are shown. All factor loadings were significant at p< 0.05. Residual variances were all significant (not shown for simplicity; details available from the authors). Residual covariances were not added to the model.


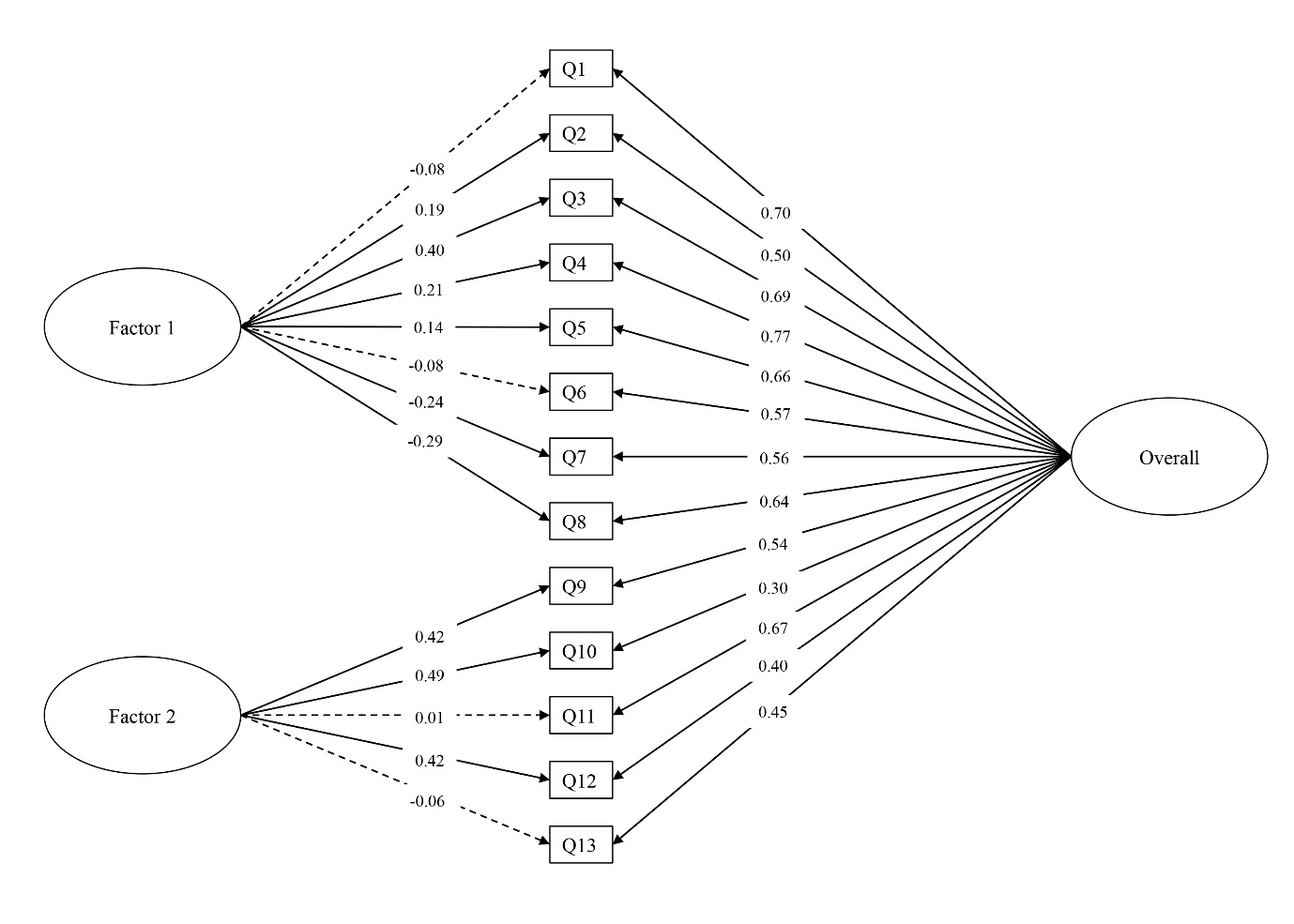


**Supplementary Figure 2.** CFA results of the alternative bifactor model (2, 3). Standardized factor loadings are shown. Non-signifcant (p≥ 0.05) factor loadings are denoted by dotted lines. Residual variances were all significant (not shown for simplicity; details available from the authors). Residual covariances were not added to the model.

**References**

1. Caballero FF, Miret M, Power M, Chatterji S, Tobiasz-Adamczyk B, Koskinen S, et al. Validation of an instrument to evaluate quality of life in the aging population: WHOQOL-AGE. Health Quality of Life Outcomes. 2013;11:177.

2. Lin C-Y, Wang J-D, Liu L-F. Can we apply WHOQOL-AGE to Asian population? Verifying its factor structure and psychometric properties in a convenience sample from Taiwan. Frontiers in Public Health. 2020;8:575374.

3. Santos D, Abad FJ, Miret M, Chatterji S, Olaya B, Zawisza K, et al. Measurement invariance of the WHOQOL-AGE questionnaire across three European countries. Quality of Life Research. 2018;27(4):1015-25.
